# Dimethano­lbis[4,4,5,5-tetra­methyl-2-(5-methyl­imidazol-4-yl)-2-imidazoline-1-oxyl-3-oxide-κ^2^ *O*,*N*]cobalt(II) diperchlorate

**DOI:** 10.1107/S1600536809051782

**Published:** 2009-12-04

**Authors:** Zhi Yong Gao, Hua Jie Guo, Wen Bei Zhang

**Affiliations:** a College of Chemistry and Environmental Science, Henan Normal University, Xinxiang 453002, People’s Republic of China

## Abstract

In the mononuclear title complex, [Co(C_11_H_17_N_4_O_2_)_2_(CH_3_OH)_2_](ClO_4_)_2_, the cobalt(II) atom lies on a symmetry centre and is coordinated by two *O*,*N*-bidentate ligands and two *trans*-arranged O atoms of the methanol mol­ecules in a slightly distorted octa­hedral geometry. In the crystal structure, cations and anions are linked by N—H⋯O and O—H⋯O hydrogen bonds into layers parallel to the *bc* plane.

## Related literature

For the use of organic radicals as building blocks for the construction of new materials, see: Marvilliers *et al.* (1999 ▶); Yamamoto *et al.* (2001 ▶). For related structures, see: Chang *et al.* (2009 ▶); Zhang *et al.* (2007 ▶); Omata *et al.* (2001 ▶); Fokin *et al.* (2004 ▶); Wang *et al.* (2005 ▶). For the synthesis of the title compound, see: Ullman *et al.* (1970 ▶, 1972 ▶).
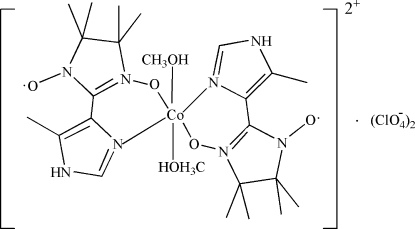


## Experimental

### 

#### Crystal data

[Co(C_11_H_17_N_4_O_2_)_2_(CH_4_O)_2_](ClO_4_)_2_
*M* *_r_* = 796.49
Triclinic, 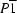

*a* = 8.761 (3) Å
*b* = 9.030 (3) Å
*c* = 11.819 (4) Å
α = 88.470 (8)°
β = 85.260 (11)°
γ = 66.638 (7)°
*V* = 855.4 (5) Å^3^
*Z* = 1
Mo *K*α radiation
μ = 0.73 mm^−1^
*T* = 295 K
0.21 × 0.10 × 0.06 mm

#### Data collection

Bruker SMART APEXII CCD area-detector diffractometer
Absorption correction: multi-scan (*SADABS*; Sheldrick, 1996 ▶) *T*_min_ = 0.862, *T*_max_ = 0.959
7639 measured reflections
3880 independent reflections
2629 reflections with *I* > 2σ(*I*)
*R*_int_ = 0.035

#### Refinement

*R*[*F* ^2^ > 2σ(*F* ^2^)] = 0.058
*wR*(*F* ^2^) = 0.169
*S* = 1.02
3880 reflections
229 parameters
H-atom parameters constrained
Δρ_max_ = 1.11 e Å^−3^
Δρ_min_ = −0.70 e Å^−3^

### 

Data collection: *SMART* (Bruker, 2002 ▶); cell refinement: *SAINT* (Bruker, 2002 ▶); data reduction: *SAINT*; program(s) used to solve structure: *SHELXS97* (Sheldrick, 2008 ▶); program(s) used to refine structure: *SHELXL97* (Sheldrick, 2008 ▶); molecular graphics: *SHELXTL* (Sheldrick, 2008 ▶); software used to prepare material for publication: *publCIF* (Westrip, 2009 ▶).

## Supplementary Material

Crystal structure: contains datablocks I, global. DOI: 10.1107/S1600536809051782/rz2401sup1.cif

Structure factors: contains datablocks I. DOI: 10.1107/S1600536809051782/rz2401Isup2.hkl

Additional supplementary materials:  crystallographic information; 3D view; checkCIF report

## Figures and Tables

**Table 1:** Hydrogen-bond geometry (Å, °)

*D*—H⋯*A*	*D*—H	H⋯*A*	*D*⋯*A*	*D*—H⋯*A*
N2—H2⋯O4	0.86	2.18	2.988 (4)	156
O8—H8*D*⋯O6^i^	0.85	1.99	2.828 (4)	167

Symmetry code: (i) 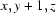.

**Fig. 1.**
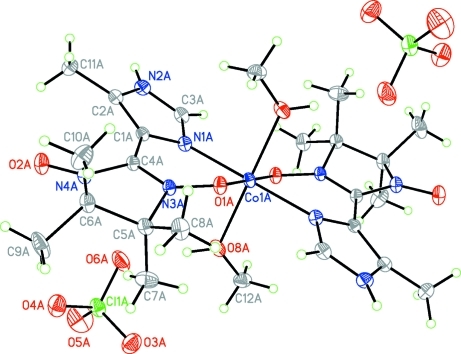
The molecular structure of the title compound with the atom-labeling scheme. Displacement ellipsoids are drawn at the 30% probability level. Unlabelled atoms are generated by the symmetry operation (-x+1,-y+1,-z+1).

## supplementary crystallographic information

### Comment

Engineering of molecular magnets constitutes a major research goal and has spawned interest in organic radicals as building blocks for construction of new materials (Marvilliers *et al.*, 1999; Yamamoto *et al.*, 2001). In this field, nitronyl nitroxides acting as useful paramagnetic building blocks have been extensively used to assemble molecular magnetic materials, because many of them are good stable spin carriers even when coordinated to metal ions. Various substitutions on radical ligands can lead to large change in not only coordination modes but also electronic behaviours, so a large number of investigations on various properties of metal-radicals complexes have been carried out and aroused intense interest and far-ranging studies (Chang *et al.*, 2009; Zhang *et al.*, 2007; Omata *et al.*, 2001; Fokin *et al.*, 2004; Wang *et al.*, 2005). In this article, we report the synthesis and crystal structure of a novel cobalt(II) complex with the nitronyl nitroxide radical.

The crystal structure of the title compound is shown in Figure. 1. The cobalt(II) ion lies on a symmetry centre and is six-coordinate in a slightly distorted octahedral CoN_4_O_2_ environment provided by two *O*,*N*-bidentate ligands and two *trans*-arranged oxygen atoms of methanol molecules. The Co1/O1/N3/C4/C1/N1 six-membered chelatating ring assumes a half-boat conformation, with atoms O1 and C1 displaced by 0.402 (3) and 0.195 (3) Å, respectively, from the mean plane of the other atoms. In the crystal structure, cations and anions are linked by N—H···O and O—H···O hydrogen bonds (Table 1) into layers parallel to the *bc* plane.

### Experimental

The nitronyl nitroxide radical (4,4,5,5-tetramethyl-2-(5-methylimidazol-4-yl)-2-imidazoline-1-oxyl-3-oxide) was synthesized according to the literature procedures (Ullman *et al.*, 1970; Ullman *et al.*, 1972). The complex was synthesized by mixing 5 ml of a methanol solution of nitronyl nitroxide radical (0.4 mmol) and 5 ml of a methanol solution of Co(ClO_4_)_2_.6H_2_O (0.2 mmol). After stirring for two hours at room temperature, the mixture solution was filtered. The clear deep purple filtrate was diffused with diethyl ether vapour at room temperature for two days, to afford deep purple crystals suitable for X-ray analysis.

### Refinement

The H atoms were positioned geometrically and refined using a riding-model approximation, with C—H = 0.93–0.96 Å, N—H = 0.86 Å, O—H = 0.85 Å, and with *U*_iso_(H) = 1.2 *U*_eq_(C, N) or 1.5 *U*_eq_(C, O) for hydroxy and methyl H atoms.

### Crystal data

**Table:**

[Co(C_11_H_17_N_4_O_2_)_2_(CH_4_O)_2_](ClO_4_)_2_	*Z* = 1
*M**_r_* = 796.49	*F*(000) = 415
Triclinic, *P*1	*D*_x_ = 1.546 Mg m^−^^3^
Hall symbol: -P 1	Mo *K*α radiation, λ = 0.71073 Å
*a* = 8.761 (3) Å	Cell parameters from 1879 reflections
*b* = 9.030 (3) Å	θ = 2.5–23.8°
*c* = 11.819 (4) Å	µ = 0.73 mm^−^^1^
α = 88.470 (8)°	*T* = 295 K
β = 85.260 (11)°	Block, purple
γ = 66.638 (7)°	0.21 × 0.10 × 0.06 mm
*V* = 855.4 (5) Å^3^	

### Data collection

**Table:**

Bruker SMART APEXII CCD area-detector diffractometer	3880 independent reflections
Radiation source: fine-focus sealed tube	2629 reflections with *I* > 2σ(*I*)
graphite	*R*_int_ = 0.035
phi and ω scans	θ_max_ = 27.5°, θ_min_ = 2.5°
Absorption correction: multi-scan (*SADABS*; Sheldrick, 1996)	*h* = −11→11
*T*_min_ = 0.862, *T*_max_ = 0.959	*k* = −11→11
7639 measured reflections	*l* = −14→15

### Refinement

**Table:**

Refinement on *F*^2^	Primary atom site location: structure-invariant direct methods
Least-squares matrix: full	Secondary atom site location: difference Fourier map
*R*[*F*^2^ > 2σ(*F*^2^)] = 0.058	Hydrogen site location: inferred from neighbouring sites
*wR*(*F*^2^) = 0.169	H-atom parameters constrained
*S* = 1.02	*w* = 1/[σ^2^(*F*_o_^2^) + (0.099*P*)^2^] where *P* = (*F*_o_^2^ + 2*F*_c_^2^)/3
3880 reflections	(Δ/σ)_max_ < 0.001
229 parameters	Δρ_max_ = 1.11 e Å^−^^3^
0 restraints	Δρ_min_ = −0.70 e Å^−^^3^

### Special details

**Table:**

Geometry. All e.s.d.'s (except the e.s.d. in the dihedral angle between two l.s. planes)are estimated using the full covariance matrix. The cell e.s.d.'s are takeninto account individually in the estimation of e.s.d.'s in distances, anglesand torsion angles; correlations between e.s.d.'s in cell parameters are onlyused when they are defined by crystal symmetry. An approximate (isotropic)treatment of cell e.s.d.'s is used for estimating e.s.d.'s involving l.s. planes.
Refinement. Refinement of *F*^2^ against ALL reflections. The weighted *R*-factor *wR* and goodness of fit *S* are based on *F*^2^, conventional *R*-factors *R* are based on *F*, with *F* set to zero for negative *F*^2^. The threshold expression of *F*^2^ > σ(*F*^2^) is used only for calculating *R*-factors(gt) *etc*. and is not relevant to the choice of reflections for refinement. *R*-factors based on *F*^2^ are statistically about twice as large as those based on *F*, and *R*-factors based on ALL data will be even larger.

### Fractional atomic coordinates and isotropic or equivalent isotropic displacement parameters (Å^2^)

**Table:**

	*x*	*y*	*z*	*U*_iso_*/*U*_eq_	
Co1	0.5000	0.5000	0.5000	0.0314 (2)	
O1	0.7069 (3)	0.5395 (3)	0.44273 (19)	0.0368 (6)	
O2	0.6974 (3)	0.6104 (4)	0.0527 (2)	0.0508 (7)	
O3	0.1971 (5)	0.1192 (5)	0.3303 (3)	0.0772 (10)	
O4	0.3181 (5)	0.0497 (4)	0.1465 (3)	0.0735 (10)	
O5	0.0366 (5)	0.1057 (6)	0.1857 (4)	0.1136 (16)	
O6	0.2364 (6)	−0.1310 (4)	0.2532 (3)	0.0897 (13)	
O8	0.3412 (3)	0.7434 (3)	0.4682 (2)	0.0479 (7)	
H8D	0.3254	0.7810	0.4015	0.072*	
N1	0.4844 (3)	0.4351 (3)	0.3332 (2)	0.0343 (6)	
N2	0.4676 (4)	0.2834 (4)	0.1975 (3)	0.0405 (7)	
H2	0.4357	0.2195	0.1628	0.049*	
N3	0.7304 (3)	0.5838 (3)	0.3387 (2)	0.0303 (6)	
N4	0.7347 (3)	0.6110 (4)	0.1542 (2)	0.0373 (7)	
C1	0.5901 (4)	0.4399 (4)	0.2393 (3)	0.0314 (7)	
C2	0.5821 (4)	0.3431 (4)	0.1536 (3)	0.0347 (7)	
C3	0.4138 (4)	0.3410 (4)	0.3035 (3)	0.0396 (8)	
H3	0.3359	0.3168	0.3498	0.048*	
C4	0.6842 (4)	0.5387 (4)	0.2443 (3)	0.0313 (7)	
C5	0.7972 (4)	0.7126 (4)	0.3165 (3)	0.0357 (8)	
C6	0.8510 (5)	0.6844 (5)	0.1892 (3)	0.0425 (9)	
C7	0.6485 (6)	0.8712 (5)	0.3436 (5)	0.0623 (12)	
H7A	0.5604	0.8808	0.2970	0.094*	
H7B	0.6817	0.9597	0.3287	0.094*	
H7C	0.6098	0.8731	0.4222	0.094*	
C8	0.9369 (5)	0.6878 (5)	0.3931 (3)	0.0502 (10)	
H8A	0.8904	0.7159	0.4698	0.075*	
H8B	0.9945	0.7551	0.3680	0.075*	
H8C	1.0136	0.5768	0.3898	0.075*	
C9	0.8249 (7)	0.8374 (6)	0.1205 (4)	0.0746 (15)	
H9A	0.8636	0.8089	0.0426	0.112*	
H9B	0.8863	0.8929	0.1506	0.112*	
H9C	0.7084	0.9065	0.1253	0.112*	
C10	1.0289 (5)	0.5579 (7)	0.1667 (4)	0.0648 (13)	
H10A	1.0382	0.4590	0.2035	0.097*	
H10B	1.1059	0.5960	0.1960	0.097*	
H10C	1.0544	0.5393	0.0864	0.097*	
C11	0.6750 (5)	0.2887 (5)	0.0407 (3)	0.0468 (9)	
H11A	0.6625	0.1935	0.0170	0.070*	
H11B	0.7911	0.2650	0.0462	0.070*	
H11C	0.6313	0.3726	−0.0139	0.070*	
C12	0.2019 (5)	0.8318 (5)	0.5434 (4)	0.0537 (10)	
H12A	0.1220	0.7833	0.5454	0.081*	
H12B	0.1516	0.9410	0.5176	0.081*	
H12C	0.2375	0.8310	0.6181	0.081*	
Cl1	0.19354 (13)	0.03724 (13)	0.22937 (9)	0.0534 (3)	

### Atomic displacement parameters (Å^2^)

**Table:**

	*U*^11^	*U*^22^	*U*^33^	*U*^12^	*U*^13^	*U*^23^
Co1	0.0307 (3)	0.0365 (4)	0.0318 (4)	−0.0185 (3)	−0.0037 (2)	0.0074 (3)
O1	0.0374 (12)	0.0511 (15)	0.0307 (12)	−0.0268 (11)	−0.0057 (10)	0.0070 (11)
O2	0.0599 (17)	0.0717 (19)	0.0359 (14)	−0.0412 (15)	−0.0107 (12)	0.0100 (13)
O3	0.099 (3)	0.089 (3)	0.061 (2)	−0.056 (2)	0.0030 (19)	−0.0138 (19)
O4	0.088 (2)	0.094 (3)	0.063 (2)	−0.064 (2)	0.0018 (18)	0.0024 (18)
O5	0.071 (2)	0.141 (4)	0.137 (4)	−0.044 (3)	−0.049 (3)	0.023 (3)
O6	0.156 (4)	0.072 (2)	0.071 (2)	−0.073 (3)	−0.032 (2)	0.0274 (18)
O8	0.0429 (14)	0.0436 (14)	0.0524 (16)	−0.0128 (12)	−0.0051 (12)	0.0156 (12)
N1	0.0322 (14)	0.0399 (16)	0.0360 (16)	−0.0196 (13)	−0.0053 (12)	0.0038 (12)
N2	0.0428 (16)	0.0409 (16)	0.0486 (18)	−0.0268 (14)	−0.0100 (14)	0.0006 (14)
N3	0.0323 (13)	0.0363 (14)	0.0296 (14)	−0.0213 (12)	−0.0038 (11)	0.0062 (11)
N4	0.0393 (16)	0.0487 (17)	0.0331 (15)	−0.0267 (14)	−0.0072 (12)	0.0069 (13)
C1	0.0313 (16)	0.0322 (17)	0.0339 (17)	−0.0157 (14)	−0.0055 (13)	0.0052 (13)
C2	0.0319 (16)	0.0374 (18)	0.0395 (19)	−0.0177 (15)	−0.0094 (14)	0.0042 (15)
C3	0.0339 (17)	0.044 (2)	0.048 (2)	−0.0229 (16)	−0.0082 (15)	0.0094 (17)
C4	0.0309 (16)	0.0363 (17)	0.0315 (17)	−0.0185 (14)	−0.0022 (13)	0.0035 (14)
C5	0.0372 (18)	0.0408 (19)	0.0399 (19)	−0.0267 (16)	−0.0063 (14)	0.0053 (15)
C6	0.048 (2)	0.057 (2)	0.039 (2)	−0.0388 (19)	−0.0089 (16)	0.0105 (17)
C7	0.061 (3)	0.044 (2)	0.088 (3)	−0.027 (2)	−0.003 (2)	−0.005 (2)
C8	0.053 (2)	0.067 (3)	0.050 (2)	−0.043 (2)	−0.0154 (18)	0.008 (2)
C9	0.108 (4)	0.085 (3)	0.069 (3)	−0.076 (3)	−0.032 (3)	0.040 (3)
C10	0.049 (2)	0.095 (4)	0.058 (3)	−0.039 (3)	0.010 (2)	−0.017 (3)
C11	0.049 (2)	0.052 (2)	0.042 (2)	−0.0218 (19)	−0.0002 (17)	−0.0075 (17)
C12	0.050 (2)	0.043 (2)	0.062 (3)	−0.0106 (19)	−0.012 (2)	−0.0047 (19)
Cl1	0.0628 (6)	0.0628 (6)	0.0512 (6)	−0.0409 (5)	−0.0164 (5)	0.0139 (5)

### Geometric parameters (Å, °)

**Table:**

Co1—O1^i^	2.042 (2)	C2—C11	1.491 (5)
Co1—O1	2.042 (2)	C3—H3	0.9300
Co1—N1^i^	2.102 (3)	C5—C8	1.524 (5)
Co1—N1	2.102 (3)	C5—C7	1.526 (6)
Co1—O8^i^	2.127 (3)	C5—C6	1.537 (5)
Co1—O8	2.127 (3)	C6—C9	1.530 (5)
O1—N3	1.308 (3)	C6—C10	1.531 (6)
O2—N4	1.271 (4)	C7—H7A	0.9600
O3—Cl1	1.430 (3)	C7—H7B	0.9600
O4—Cl1	1.441 (3)	C7—H7C	0.9600
O5—Cl1	1.402 (4)	C8—H8A	0.9600
O6—Cl1	1.439 (3)	C8—H8B	0.9600
O8—C12	1.415 (5)	C8—H8C	0.9600
O8—H8D	0.8501	C9—H9A	0.9600
N1—C3	1.302 (4)	C9—H9B	0.9600
N1—C1	1.396 (4)	C9—H9C	0.9600
N2—C3	1.343 (5)	C10—H10A	0.9600
N2—C2	1.376 (4)	C10—H10B	0.9600
N2—H2	0.8600	C10—H10C	0.9600
N3—C4	1.343 (4)	C11—H11A	0.9600
N3—C5	1.504 (4)	C11—H11B	0.9600
N4—C4	1.368 (4)	C11—H11C	0.9600
N4—C6	1.506 (4)	C12—H12A	0.9600
C1—C2	1.380 (5)	C12—H12B	0.9600
C1—C4	1.440 (4)	C12—H12C	0.9600
			
O1^i^—Co1—O1	180.000 (1)	N4—C6—C9	109.6 (3)
O1^i^—Co1—N1^i^	88.12 (10)	N4—C6—C10	107.2 (3)
O1—Co1—N1^i^	91.88 (10)	C9—C6—C10	111.2 (4)
O1^i^—Co1—N1	91.88 (10)	N4—C6—C5	100.7 (3)
O1—Co1—N1	88.12 (10)	C9—C6—C5	114.9 (4)
N1^i^—Co1—N1	180.0	C10—C6—C5	112.4 (3)
O1^i^—Co1—O8^i^	91.60 (10)	C5—C7—H7A	109.5
O1—Co1—O8^i^	88.40 (10)	C5—C7—H7B	109.5
N1^i^—Co1—O8^i^	90.41 (11)	H7A—C7—H7B	109.5
N1—Co1—O8^i^	89.59 (11)	C5—C7—H7C	109.5
O1^i^—Co1—O8	88.40 (10)	H7A—C7—H7C	109.5
O1—Co1—O8	91.60 (10)	H7B—C7—H7C	109.5
N1^i^—Co1—O8	89.59 (11)	C5—C8—H8A	109.5
N1—Co1—O8	90.41 (11)	C5—C8—H8B	109.5
O8^i^—Co1—O8	180.00 (14)	H8A—C8—H8B	109.5
N3—O1—Co1	122.87 (18)	C5—C8—H8C	109.5
C12—O8—Co1	122.0 (2)	H8A—C8—H8C	109.5
C12—O8—H8D	109.8	H8B—C8—H8C	109.5
Co1—O8—H8D	122.5	C6—C9—H9A	109.5
C3—N1—C1	105.6 (3)	C6—C9—H9B	109.5
C3—N1—Co1	126.3 (2)	H9A—C9—H9B	109.5
C1—N1—Co1	125.3 (2)	C6—C9—H9C	109.5
C3—N2—C2	109.0 (3)	H9A—C9—H9C	109.5
C3—N2—H2	125.5	H9B—C9—H9C	109.5
C2—N2—H2	125.5	C6—C10—H10A	109.5
O1—N3—C4	126.8 (3)	C6—C10—H10B	109.5
O1—N3—C5	120.2 (2)	H10A—C10—H10B	109.5
C4—N3—C5	112.6 (3)	C6—C10—H10C	109.5
O2—N4—C4	125.5 (3)	H10A—C10—H10C	109.5
O2—N4—C6	123.4 (3)	H10B—C10—H10C	109.5
C4—N4—C6	111.0 (3)	C2—C11—H11A	109.5
C2—C1—N1	109.8 (3)	C2—C11—H11B	109.5
C2—C1—C4	131.0 (3)	H11A—C11—H11B	109.5
N1—C1—C4	119.2 (3)	C2—C11—H11C	109.5
N2—C2—C1	104.0 (3)	H11A—C11—H11C	109.5
N2—C2—C11	121.3 (3)	H11B—C11—H11C	109.5
C1—C2—C11	134.4 (3)	O8—C12—H12A	109.5
N1—C3—N2	111.6 (3)	O8—C12—H12B	109.5
N1—C3—H3	124.2	H12A—C12—H12B	109.5
N2—C3—H3	124.2	O8—C12—H12C	109.5
N3—C4—N4	107.6 (3)	H12A—C12—H12C	109.5
N3—C4—C1	126.3 (3)	H12B—C12—H12C	109.5
N4—C4—C1	126.1 (3)	O5—Cl1—O3	111.2 (3)
N3—C5—C8	109.8 (3)	O5—Cl1—O6	110.4 (3)
N3—C5—C7	105.0 (3)	O3—Cl1—O6	109.6 (2)
C8—C5—C7	111.4 (3)	O5—Cl1—O4	109.4 (3)
N3—C5—C6	100.1 (3)	O3—Cl1—O4	108.1 (2)
C8—C5—C6	115.5 (3)	O6—Cl1—O4	108.0 (2)
C7—C5—C6	113.8 (3)		
			
N1^i^—Co1—O1—N3	−151.2 (2)	C5—N3—C4—N4	8.1 (4)
N1—Co1—O1—N3	28.8 (2)	O1—N3—C4—C1	4.0 (5)
O8^i^—Co1—O1—N3	118.5 (2)	C5—N3—C4—C1	−168.7 (3)
O8—Co1—O1—N3	−61.5 (2)	O2—N4—C4—N3	−172.0 (3)
O1^i^—Co1—O8—C12	39.5 (3)	C6—N4—C4—N3	11.4 (4)
O1—Co1—O8—C12	−140.5 (3)	O2—N4—C4—C1	4.7 (6)
N1^i^—Co1—O8—C12	−48.7 (3)	C6—N4—C4—C1	−171.8 (3)
N1—Co1—O8—C12	131.3 (3)	C2—C1—C4—N3	−155.3 (3)
O1^i^—Co1—N1—C3	−25.0 (3)	N1—C1—C4—N3	25.4 (5)
O1—Co1—N1—C3	155.0 (3)	C2—C1—C4—N4	28.5 (6)
O8^i^—Co1—N1—C3	66.6 (3)	N1—C1—C4—N4	−150.8 (3)
O8—Co1—N1—C3	−113.4 (3)	O1—N3—C5—C8	41.8 (4)
O1^i^—Co1—N1—C1	177.2 (3)	C4—N3—C5—C8	−145.0 (3)
O1—Co1—N1—C1	−2.8 (3)	O1—N3—C5—C7	−78.0 (4)
O8^i^—Co1—N1—C1	−91.2 (3)	C4—N3—C5—C7	95.2 (4)
O8—Co1—N1—C1	88.8 (3)	O1—N3—C5—C6	163.8 (3)
Co1—O1—N3—C4	−34.9 (4)	C4—N3—C5—C6	−23.0 (3)
Co1—O1—N3—C5	137.3 (2)	O2—N4—C6—C9	36.9 (5)
C3—N1—C1—C2	−1.0 (4)	C4—N4—C6—C9	−146.5 (4)
Co1—N1—C1—C2	160.5 (2)	O2—N4—C6—C10	−83.8 (4)
C3—N1—C1—C4	178.4 (3)	C4—N4—C6—C10	92.7 (4)
Co1—N1—C1—C4	−20.1 (4)	O2—N4—C6—C5	158.4 (3)
C3—N2—C2—C1	−0.7 (4)	C4—N4—C6—C5	−25.0 (4)
C3—N2—C2—C11	174.6 (3)	N3—C5—C6—N4	26.4 (3)
N1—C1—C2—N2	1.0 (4)	C8—C5—C6—N4	144.2 (3)
C4—C1—C2—N2	−178.3 (3)	C7—C5—C6—N4	−85.1 (3)
N1—C1—C2—C11	−173.3 (4)	N3—C5—C6—C9	144.0 (3)
C4—C1—C2—C11	7.4 (6)	C8—C5—C6—C9	−98.1 (4)
C1—N1—C3—N2	0.6 (4)	C7—C5—C6—C9	32.5 (4)
Co1—N1—C3—N2	−160.7 (2)	N3—C5—C6—C10	−87.4 (3)
C2—N2—C3—N1	0.0 (4)	C8—C5—C6—C10	30.4 (4)
O1—N3—C4—N4	−179.2 (3)	C7—C5—C6—C10	161.1 (3)

Symmetry codes: (i) −*x*+1, −*y*+1, −*z*+1.

### Hydrogen-bond geometry (Å, °)

**Table:**

*D*—H···*A*	*D*—H	H···*A*	*D*···*A*	*D*—H···*A*
N2—H2···O4	0.86	2.18	2.988 (4)	156
O8—H8D···O6^ii^	0.85	1.99	2.828 (4)	167

Symmetry codes: (ii) *x*, *y*+1, *z*.
